# Recent Observations of Human-induced Asymmetric Effects on Climate in Very High-Altitude Area

**DOI:** 10.1371/journal.pone.0081535

**Published:** 2014-01-29

**Authors:** Heli Lu, Guifang Liu

**Affiliations:** 1 Institute of Natural Resources and Environmental Science, and College of Environment and Planning, Henan University, Kaifeng, China; 2 Henan Collaborative Innovation Center for Coordinated Developments in Central China Economic Zone, Zhengzhou, China; 3 United Nations University-Institute of Advanced Studies, Yokohama, Japan; University of Oxford, United Kingdom

## Abstract

Like urban heat islands (UHI), human-induced land degradation (HLD) is a phenomenon attributed to human activities, but this phenomenon occurs in non-urban areas. Although a large body of work has demonstrated that land-cover change influences local climate systems, little work has been done on separating the impact of HLD from naturally-occurring fluctuations in very high-altitude areas. We developed an innovative NDVI-difference method in order to evaluate HLD effects upon the climate system in the central Tibet Plateau. The results show that the minimum temperature increased at a significantly faster pace than the maximum temperature in the growing season at HLD meteorological stations, but this was reversed at stations with natural forces only. Further analysis revealed that abrupt changes of minimum temperature occurred five years earlier and amplitudes of these changes were 1.4 times larger than at stations with natural forces only. Therefore, our results complement other evidence that points to the fact that local effects from UHI contribute to climatic asymmetry observed between minimum and maximum temperature trends. Accordingly, we stress the need for consideration of non-urban factors from anthropogenic activities, such as human-induced land degradation, in understanding these asymmetric diurnal changes.

## Introduction

Human activities, such as burning fossil fuels, releasing chemicals into the atmosphere from industrial activities, reducing the amount of forest cover, and rapid expansion of farming, are changing the balance of the climate system [1]–[7]. A prominent example is that that the rise of the minimum temperature has occurred at a higher rate than the rise of the maximum temperature since 1950, resulting in a broad decline in the diurnal temperature range (DTR) [8]–[10]. A large body of work has demonstrated that land-cover change provides an additional major forcing of the climate, through changes in the physical properties of the land surface [11]–[14]. Since land degradation is a human-induced process leading to different amounts and rates of climate system variability, a pervasive issue in this topic has been how to separate human-induced climate change from natural climate fluctuations and henceforth help to quantify human influences on global warming [15]–[20], [3]. Very high-altitude areas are ideal environments for monitoring human-induced climate change because they have low temperature conditions. However, human influences on the climatic system at high altitudes have a high degree of complexity and a high degree of uncertainty. Difficulties in taking direct measurements in very high altitude areas make this an even more challenging consideration.

Human-induced land degradation (HLD) is a man-made phenomenon that negatively affects the effective function of land within an ecosystem, to accept, store and recycle water, energy, and nutrients [21]–[23]. Changes in satellite-measured greenness are a good indicator of land degradation. The normalized different vegetation index (NDVI), which is a non-linear transformation of the visible (red) and near-infrared bands from remote sensing, has been proven to be capable of highlighting the area with comparatively reduced vegetation activity and thus monitoring the spatial- pattern and magnitude of land degradation [24]–[29]. In this study an innovative NDVI-difference method that utilizes spatially distributed information of time-series NDVI in the growing season, with verification of the actual vegetation conditions, was developed in order to evaluate HLD effects upon climate in the central Tibet Plateau (TP), where long-term pressures resulting from growing human populations and higher numbers of grazing livestock had been putting the actual and/or future capability of land in danger [30]. The Mann–Kendall (MK) test was used for detecting temperature trends and abrupt changes in time series.

## Background and Methodology

There are clear suggestions from a number of high-altitude climate records from this century that the amplitude of temperature changes at high altitudes is greater than the observed global or hemispheric change; furthermore, a number of lines of evidence suggest that the warming signal in the tropics during the past few decades increases with height. The IPCC Second Assessment Report [31], in its chapter on the impacts of climate change on mountainous regions [32], has recommended that “future research needed to understand and predict effects of climatic change on mountain regions should represent balance and coordination between field studies, monitoring, experimental studies, and modeling.”

The Tibetan Plateau (TP), with the most prominent and complex terrain on the globe and an elevation averaging more than 4000 m above mean sea level [33], is often called the “Third Pole” because its geographic significance is akin to that of Antarctica and the Arctic [34]. The TP plays an important role in global atmospheric circulation through orographic and thermal forcing mechanisms. The TP, which is considered to be “the driver and amplifier of global climate change,” is the ‘start-up region’ for climate change in China, as well as for the world [35].

There is evidence that the degradation of grassland has become increasingly serious with growing population and livestock in recent years. Human-induced land degradation has become a widespread problem in the TP, with its large area and wide-ranging types of degradation. It was estimated that the total area of degraded land in the TP was approximately 4.0×10^7^ to 6.0×10^7^ ha in 1990s [36]. Serious degradation is particularly found around water sites and the camps where animals gather in the winter-spring. The causes of land degradation include inappropriate human activities like overgrazing, an imbalanced use of grasslands between winter-spring and summer –autumn and poor management of grasslands.

### NDVI-difference method

For global change research the long term Global Inventory Model and Mapping Studies (GIMMS) NDVI dataset provides a critical historical perspective on vegetation changes related to anthropogenic and/or natural causes (http://glcf.umd.edu/data/gimms/). Tests of GIMMS data against measures of vegetation and climate have shown that GIMMS data are able to capture general patterns of vegetation, inter-annual variations of vegetation, and climate signals [27]. Here we use the bi-monthly Global Inventory Modeling and Mapping Studies (GIMMS) Satellite Drift Corrected and NOAA-16 incorporated 8 km NDVI in mainland China from 1982 to 2006.

To minimize the influence of non-vegetation land-cover, NDVI values below 0.1, representing snow, inland water bodies, desert and exposed soil, were removed. Furthermore NDVI was only calculated in growing season in order to reduce other factors not related to actual vegetation change. NDVI was sampled for 8×8 pixel windows centered on each meteorological station involved in this study. Such a pixel window, with a radius of about 32 km, was chosen because it is generally accepted that meteorological station observation data is influenced by mesoscale climate (1 km to 30 km) including proximity and size of vegetation cover types, urbanized areas and other factors [28], [29]. It is assumed that each station NDVI is comprised of natural components and anthropogenic components due to averaging the corresponding 8×8 pixel window. For example, interannual changes in climate factors, especially temperature and precipitation, can profoundly influence vegetation growth and vegetation cover and are thus related to changes on NDVI. On the other hand, clearing of forest for agriculture also influences NDVI. The basic aim is to remove the climate signal in order to isolate the signal of human activities. We assume that NDVI in the 128 km radius area (32×32 pixel window) around the station should not be sensitive to the HLD effect on the station. Hence natural components could be derived from the 32×32 pixel window. Consequently NDVI difference, which is the 8×8 pixel window minus the 32×32 pixel window, should be the best indicator available, in which a decline trend indicates intensity of human activities and occurrence of the HLD process. In particular, nightlights data from the Defense Meteorological Satellite Program Operational Linescan System (DMSP-OLS) (NOAA's National Geophysical Data Center) sensor was used to separate HLD stations from stations of urban expansion, which leads to a similar NDVI reduction. Finally HLD stations were identified by following the steps:

Step 1. NDVI difference values around the station during growing season was aggregated and averaged for each year. Ordinary Least Squares (OLS) regression technique based on a linear regression model (Y = a+bX+ε) was then applied on the dataset.

Step 2. Each 1 km grid in the satellite image of the DMSP-OLS data above the nightlights intensity threshold was classified into urban and non-urban categories. The decision tree to categorize the stations was based on analysis of the grid box group within 32 km of each station [15].

Step 3. Only stations with negative trend and no more than 25% urban grid boxes were classified as HLD.

Step 4. Results were validated through the auxiliary data of Landsat TM satellite image with 30 m spatial resolution from the United States Geological Survey (http://www.usgs.gov/).

No specific permissions were required for these locations/activities, and the field studies did not involve endangered or protected species.

### Identification of growing season

A number of methods, including NDVI threshold, lowest NDVI value from derivative, smoothing algorithms and model fit, have been devised for determining vegetation phenological stage [24], [37]. In this study the model fit method, which was developed by Jönsson and Eklundh [37] for analyzing time-series NDVI data, was selected to indentify the growing season. It implements three processing methods based on least-squares fits to the upper envelope of the NDVI data: asymmetric Gaussian (AG), double logistic (DL) and adaptive Savitzky-Golay filtering (SG). The benefit of both asymmetric Gaussian and double logistic approaches is that they are less sensitive to incomplete time-series data with data gaps. In contrast, whilst adaptive Savitzky-Golay filtering is sensitive to data gaps, it can capture subtle and rapid changes in the time-series. Since the GIMMS data have received even better corrections for noise and artifacts [38], [39] our method starts with SG to fit the time-series data. To smooth data and suppress disturbances, the SG method uses a filter, and replaces y_i_ with a linear combination of nearby values in a window:
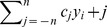
(1)where y_i_ are each data value, c_j_ are weights, i = 1, …, n. For each value of y_i_, the quadratic polynomial f (t) = c1+c2t+c3t2 was fit to all 2n+1 points in the moving window and the value y_i_ was replaced with the value of the polynomial at position t_i_.

The beginning of a season is defined from the SG functions as the point in time when the value has increased by 10% of the distance between the left minimum level and the maximum. The end of the season is defined in a similar way. As such the growing season is defined as the difference between the beginning and the end of a season.

### Climatic Zones

The complex topography of the TP makes it difficult to analyze climate change at the scale of the whole region. Therefore, the TP is divided into different climatic zones in order to identify changes more clearly and accurately. The method, based on Lin and Wu [40], uses temperature and precipitation as the basic indicators (Table 1). The TP is divided into different climatic zones according to (1) the number of days per year on which the average temperature is above 10°C, with average temperature in the hottest month as an auxiliary indicator, and (2) an aridity index (Eq. 1), with annual precipitation as an auxiliary indicator:
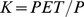
(2)where *K* is the aridity index, *PET* is potential evapotranspiration and *P* is annual precipitation.

**Table 1:** (A) Temperature and (B) precipitation indicators for climatic regionalization of the Tibetan Plateau (TP), listing the number of days per year on which average temperature is above 10°C, the average temperature in the hottest month, the aridity index, and the annual precipitation [40].

(A) Temperature		
Type	Days above 10°C	Temperature in hottest month (°C)
Subtropical	>180	>18
Plateau temperate	180 to 50	17.9 to 12
Plateau subfrigid	<50	11.9 to 6
Plateau frigid	0	<6

(B) Precipitation		
Type	Aridity index	Annual precipitation (mm)
Humid	<1.0	>800
Semi-humid	1.0 to 1.5	800 to 500
Semi-arid	1.6 to 5.0	499 to 200
Arid	5.1 to 15.0	199 to 50
Extremely arid	>15.0	<50

**Figure 1:**
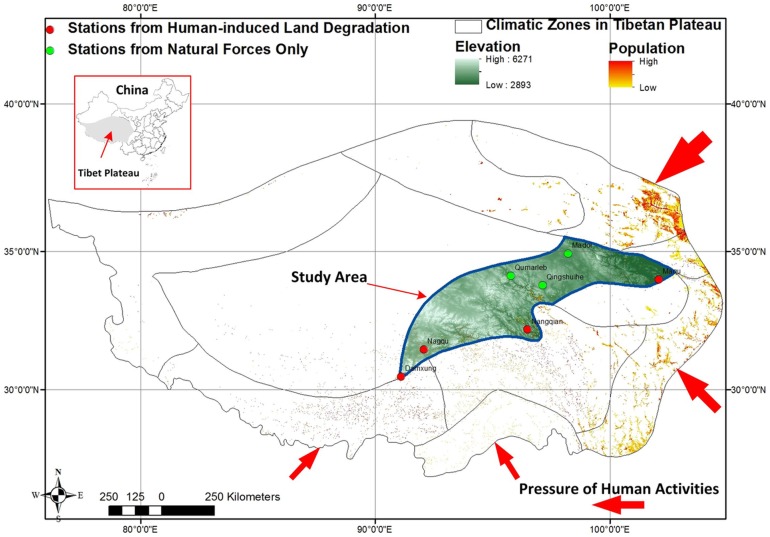
Study area. The study area is located in the central Tibet Plateau, with 7 stations in the same climatic zone. Distribution of population is a reasonable indicator for pressures from human activities. Stations in areas of human-induced land degradation (Group B including Damxung, Maqu, Nangqian and Nagqu) are close to high-pressure areas, while stations in areas of natural forces only (Group A including Qumarleb, Madoi and Qingshuihe) are located in the sparsely populated hinterland.

Based on the temperature and precipitation indicators, the TP is divided into 11 climatic zones (Table 2, Fig. 1).

**Table 2:** The 11 climatic zones of the Tibetan Plateau (shown in Fig. 1) based on the indicators given in Table 1, and average annual temperature for each zone for 1961–2005.

Zone	Type		Temperature (°C)
	Temperature	Precipitation	
1	Plateau temperate	Extremely arid	3.62
2	Plateau temperate	Semi-arid	3.59
3	Plateau temperate & frigid	Arid	0.55
4	Plateau subfrigid	Semi-humid	−3.60
5	Plateau subfrigid	Semi-humid	4.77
6*	Plateau subfrigid	Semi-humid	−0.66
7	Plateau temperate	Humid	8.26
8	Plateau subfrigid	Humid	0.14
9	Plateau temperate	Semi-humid	5.71
10	Plateau temperate	Semi-arid	4.64
11	Subtropical	Humid	14.56

* Research area.

### Mann-Kendall test

The Mann-Kendall test is a non-parametric statistical procedure that is well suited to analyzing trends in data over time [41]. One advantage of this test is that the data need not conform to any particular distribution. The second advantage of the test is its low sensitivity to abrupt breaks due to inhomogeneous time series [42]. As a result, the time series of DTR values at seven sites were analyzed for monotonous increasing or decreasing trends with the Mann-Kendall test.

The Mann-Kendall test can be viewed as a nonparametric test for zero slope of the first-order regression of time-ordered data versus time. Each data value is compared with all subsequent data values. If a data value from a later time period is higher than a data value from an earlier time period, the statistic S is incremented by 1. On the other hand, if the data value from a later time period is lower than a data value sampled earlier, S is decremented by 1. The net result of all such increments and decrements yields the final value of S [43]. The MK test used to be applied by considering the statistic S as [44]:
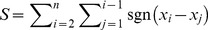
(3)where x_j_ is the sequential data values, n is the length of the series and 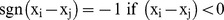; 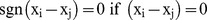 and 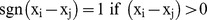.

The standard test statistic Z is computed as follows:
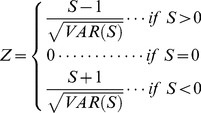
(4)


The presence of a statistically significant trend is evaluated using the Z value. A positive (negative) value of Z indicates an upward (downward) trend.

The Sen slope [41] calculation is determined along with the Mann-Kendall test. Similar to Mann-Kendall test, the advantage of Sen's slope estimator is that it is insensitive to outliers or missing data. Therefore it is more rigorous than the usual regression slopes and thus provides a realistic measure of the trends in the data series.

Simply speaking, Sen's slope is the median of all differences between successive data values. The magnitude of the trend is computed as [45], [46]:
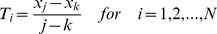
(5)where T_i_ is the slope of all data pairs, and x_j_ and x_k_ are data values at times j and k (j>k) correspondingly.

The estimate of the slope of the trend is calculated as:
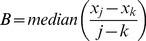
(6)


A positive value of B means an increasing trend whereas a negative value means a decreasing trend.

The sequential version of the Mann–Kendall test was used to detect abrupt changes in climate against the long-term trend [47]. The null hypothesis H0 presumes that the sample under investigation shows no indication of a developing trend. This rank-based test considers the relations between all terms in the time series (×1, ×2, ×3,…×n). The following steps are applied in order to accept or reject the null hypothesis [48]:

#### Step 1. Definition of the test statistic

The test statistic t_j_ variables are computed as follows:
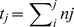
(7)where nj denotes for each element x_j_ (j>k) the number of cases where x_j_>x_k_, with j = 1,2,…..n and k = 1,2,…..j−1. The distribution of t_j_ is asymptotically normal with E(tj) = [j(j−1)]/4 and Var(tj) = [j(j−1)(2j+5)]/72.

#### Step 2. Calculation of the reduced variables

A reduced variable, called statistic u(t),is calculated for each of the test statistic variables t_j_ as follows:
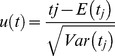
(8)


#### Step 3. Determination of the starting of an emerging trend

Similarly to the calculation of progressive rows of statistic u(t),the retrograde rows of statistic u'(t) are computed backward starting from the end of a series. The intersection point of the progressive and retrograde rows of the statistic u(t) and u'(t) provides the point in time of the beginning of an emerging trend within the time series. The null hypothesis H0 should be rejected when at least one of the reduced variables is greater than a chosen level of significance of the Gaussian distribution.

### Meteorological data

Minimum and maximum temperatures from a total of 7 national meteorological stations for the period from 1 January 1982 to 31 December 2006 were used for this study. The observational data are derived from the “Daily Surface Climate Variables of China” catalog issued by the National Meteorological Information Center of the China Meteorological Administration (NMIC/CMA). The station locations span from 30.5°N to 34.9°N, and from 91.1°E to 102.1°E, whilst the altitude of the stations varies from 3471.4 m above sea level (a.s.l.) to 4507 m a.s.l. (Table 3).

**Table 3:** List of selected stations in the Tibetan Plateau (TP) included in the analysis, listed with World Meteorological Organization (WMO) number, station name, latitude, longitude and elevation.

WMO number	Station name	North latitude	East longitude	Elevation (m)
55299	Nagqu	31.5	92.1	4507.0
56074	Maqu	34.0	102.1	3471.4
56125	Nangqian	32.2	96.5	3643.7
55493	Damxung	30.5	91.1	4200.0
56021	Qumarleb	34.1	95.8	4175.0
56033	Madoi	34.9	98.2	4272.3
56034	Qingshuihe	33.8	97.1	4415.4

Quality control (QC) of observational data is an extremely important factor in evaluating human-induced effects on climate in the study area. The QC must be undertaken prior to any data analysis to eliminate erroneous values. For example corrections must be performed on meteorological observed data if station metadata (coordinates, elevation, etc) change. In recent years researchers have paid more attention to atmospheric data assimilation techniques that could improve QC [49], [50].

There were two stages of QC in this study. Firstly, the data were quality controlled by the provider of NMIC/CMA. They were checked by Feng et al. [51] for homogeneity and consistency in five tiers: high–low extreme checking for daily values, internal consistency checking, temporal outlier checking, spatial outlier checking and missing data checking. The data were then screened with the software package C3 extraQC (http://www.c3.urv.cat/data.html): an expanded version of RClimDex (http://etccdi.pacificclimate.org/software.shtml, running with R 1.84 or a later version) http://cccma.seos.uvic.ca/ETCCDMIin order to (1) identify errors in the minimum and maximum temperature; (2) search for outliers in the minimum and maximum temperature; (3) use the generalized data plot to visually inspect the data and (4) assess data homogeneity.

## Results and Analysis

**Figure 2:**
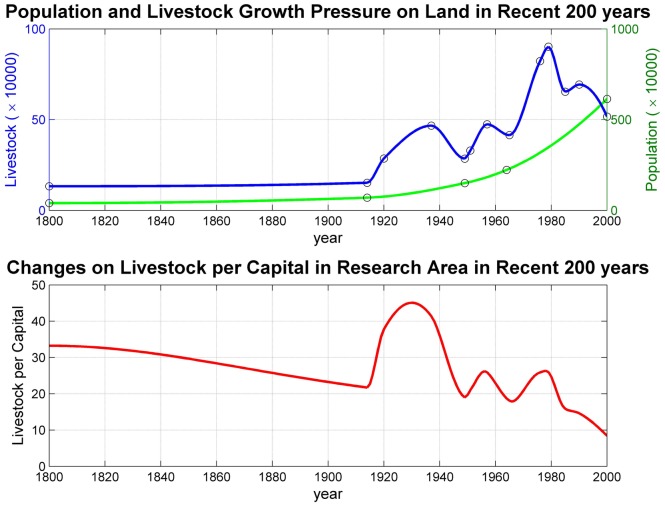
Historical land degradation. Land degradation can be mapped and observed in physical terms, but can only be explained and understood when hidden social, political, and economic structures are analyzed [57]. Socioeconomic data from the 200 years from 1800 to 2000 were collected to explore the reasons for human-induced land degradation. It is clear that growing population and livestock have been putting high pressure on the study area. The flattening curve of population has changed since the 1950s and increased dramatically since the 1980s. Such growth is attributed by People's Republic of China officials to the improved quality of health and lifestyle of the average Tibetan since the beginning of reforms under the Chinese governance. In contrast, population livestock reached its climax in 1980 and has steadily declined since then, resulting in a downward trend in livestock per capita. Loss and degradation of grassland due to increasing human population and livestock pressures are the principal threats to this extremely fragile zone.

**Figure 3:**
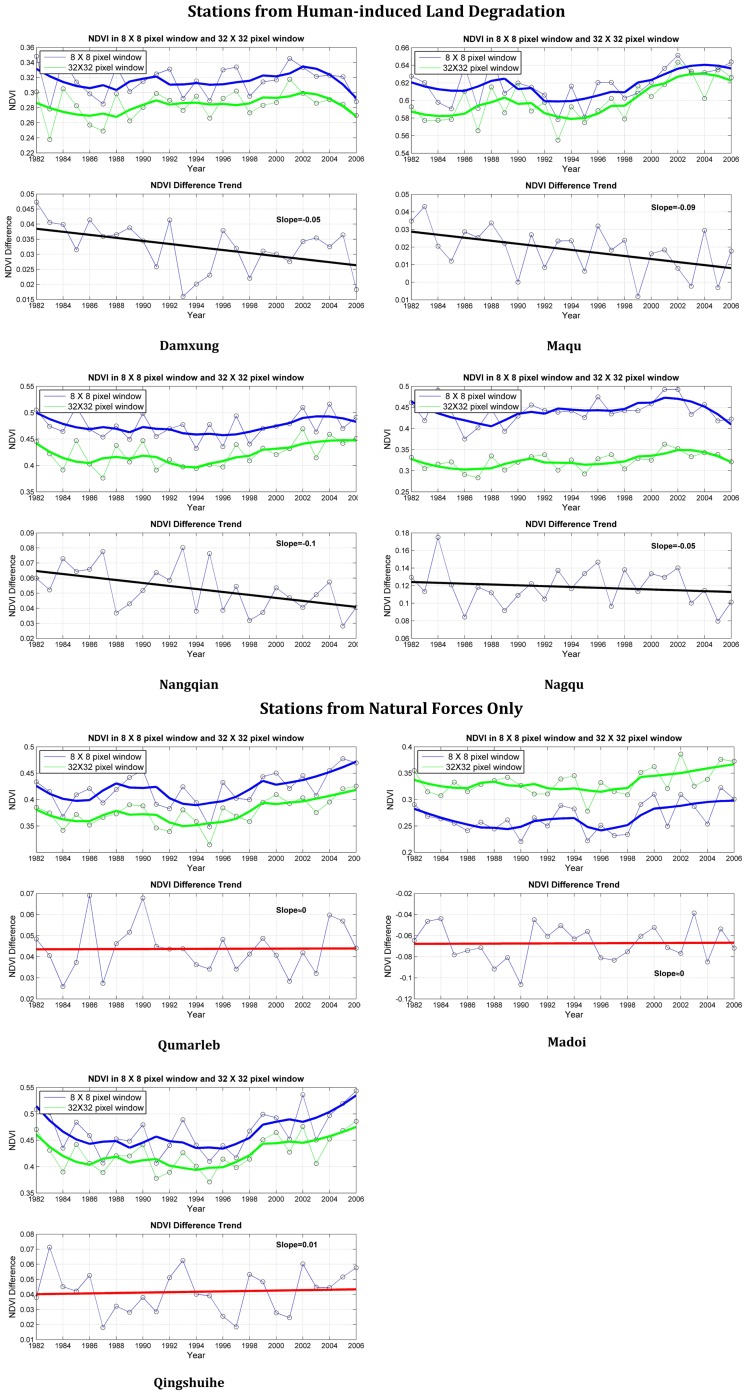
NDVI difference. Time-series NDVIs in 8×8 (32 km) and 32×32 (128 km) pixel windows of the stations are shown at the top. Heavy lines are the result of smoothing with Savitzky-Golay filtering. Time-series NDVI differences and their trends are shown on the bottom. Zero values of trends from stations in areas with natural forces only (Group A) indicate that there are almost no differences between 32 km areas and 128 km areas, since no additional forces influence the vegetation except natural forces. Negative values of trends from stations in HLD areas (Group B) indicate that NDVI decreases more in the 32 km areas than in the 128 km areas, since pasture degradation from human activities occurred close to these stations. Note that there are two opposite initial states for NDVI difference: 32×32 pixel window above 8×8 pixel window at Madoi station and 8×8 pixel windows above 32×32 pixel window at other stations.

**Figure 4:**
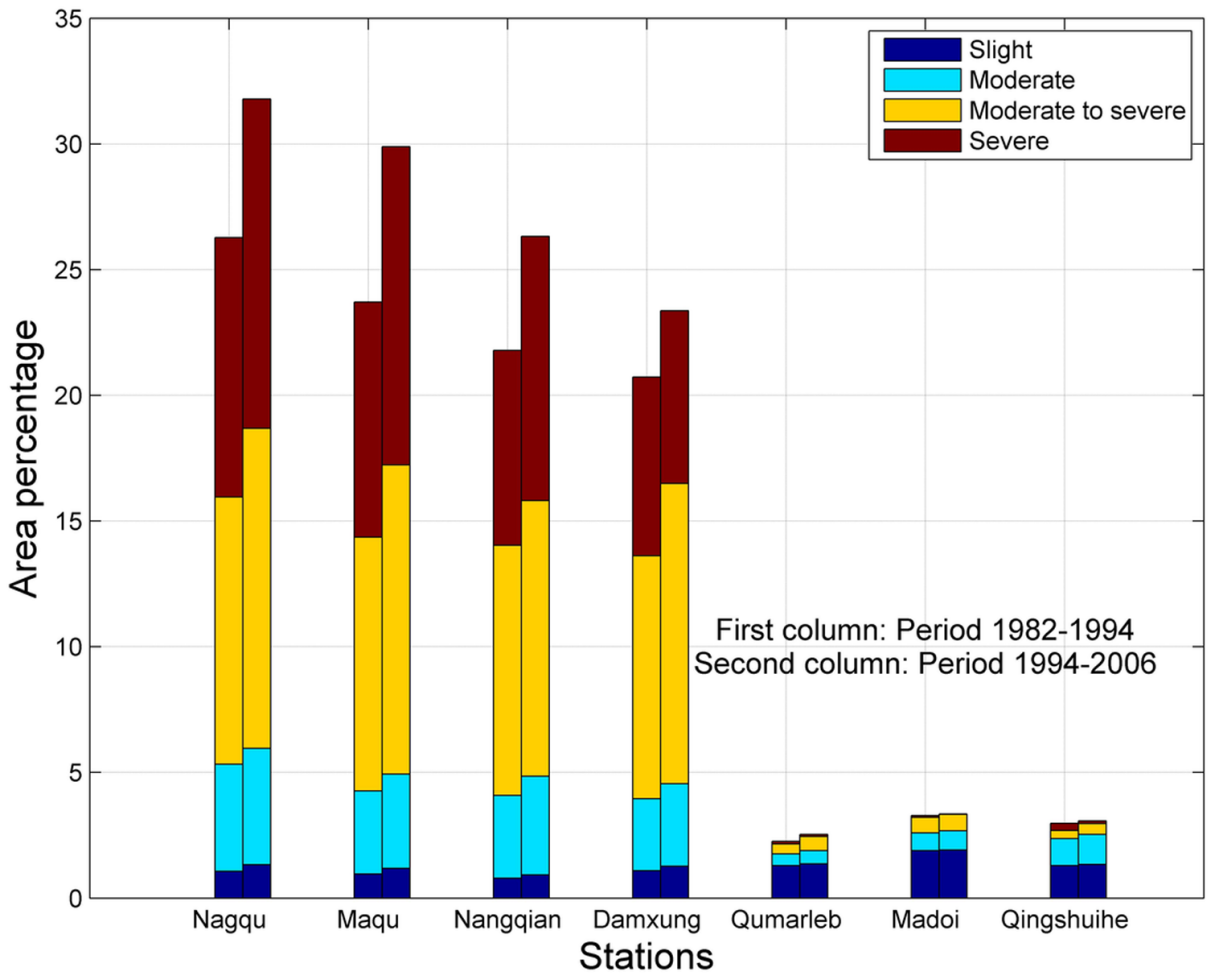
Degraded land from high resolution satellite images. We used 32(TM) satellite images with 30 m spatial resolution during two periods of 1982–1994 and 1994–2006 downloaded from U.S. Geological Survey (USGS), which are centered on each station. Four ranks of degraded land were determined through remote sensing interpretation: slight, moderate, moderate to severe and severe. The major steps included (1) field survey for interpretation and mapping; (2) image preprocessing, including geometric rectification, image registration and atmospheric correction; (3) classification using visual interpretation of spectral features and texture types; and (4) final mapping after validation. It can be clearly seen that land degradation rate is high due to the pressure of human activities at HLD stations. For instance, total degraded area increased 5.6% in the period of 1994–2006 in Nagqu. The part that increased the most was the rank of severe degradation, about 51% of total degraded area. This result confirmed the declining trends of NDVI.

Seven meteorological stations with an average altitude of around 4000 m in the same climatic zone in the central Tibet Plateau were classified into two groups: stations in areas of natural forces only (Group A) and stations in areas of HLD (Group B) (Fig. 1) (Fig. 2). There is a transition zone between the area that has a high-pressure exerted from human activities (close to Group B) and the area with a low-pressure (close to Group A). Although NDVI differences trends in Group A remained unchanged, those in Group B showed clear declining trends (Fig. 3). The land degradation for stations were also visually interpreted and confirmed through the auxiliary data of Landsat TM satellite image with 30 m spatial resolution in the two periods of 1982–1994 and 1994–2006 (Fig. 4). Wessels [52] proposed the use of RESTREND (residual trend between actual NDVI and predicted NDVI) to distinguish land degradation from the effects of rainfall variability in South Africa. We use the RESTREND technique to validate the NDVI-difference method presented in this study. NDVI data and precipitation during the growing season of 1982–2006 are used in this technique and a comparison of results from NDVI-difference method and RESTREND is shown in Table 4. It can be seen that same results could be concluded for the 4 stations of Damxung, Qumarleb, Madoi and Qingshuihe, but the 3 stations of Nagqu, Maqu and Nangqian were not available using RESTREND. The reason may be that RESTREND is less sensitive to human-induced land degradation in alpine meadow in very high-altitude area of TP than NDVI-difference method, since RESTREND developed by Wessels was originally used in semi-arid Karoo, Southern Africa.

**Table 4:** A comparison of results from NDVI-difference method and RESTREND.

Site Name	NDVI-difference method			RESTREND		
	NDVI-difference trend	Validation from high resolution satellite images	Result	R-square statistic	slope	Result
***Nagqu***	***−0.05***	***√***	Human-induced land degradatio***n***	***<0.1***	***N/A***	***N/A***
***Maqu***	***−0.09***	***√***	Human-induced land degradatio***n***	***<0.1***	***N/A***	***N/A***
***Nangqian***	***−0.1***	***√***	Human-induced land degradatio***n***	***<0.1***	***N/A***	***N/A***
***Damxung***	***−0.05***	***√***	Human-induced land degradatio***n***	***0.15***	***−0.13***	Human-induced land degradation
Qumarleb	zero	***√***	Natural forces only	0.22	0.2	Natural forces only
Madoi	zero	***√***	Natural forces only	0.12	0.13	Natural forces only
Qingshuihe	0.01	***√***	Natural forces only	0.11	0.17	Natural forces only

**Figure 5:**
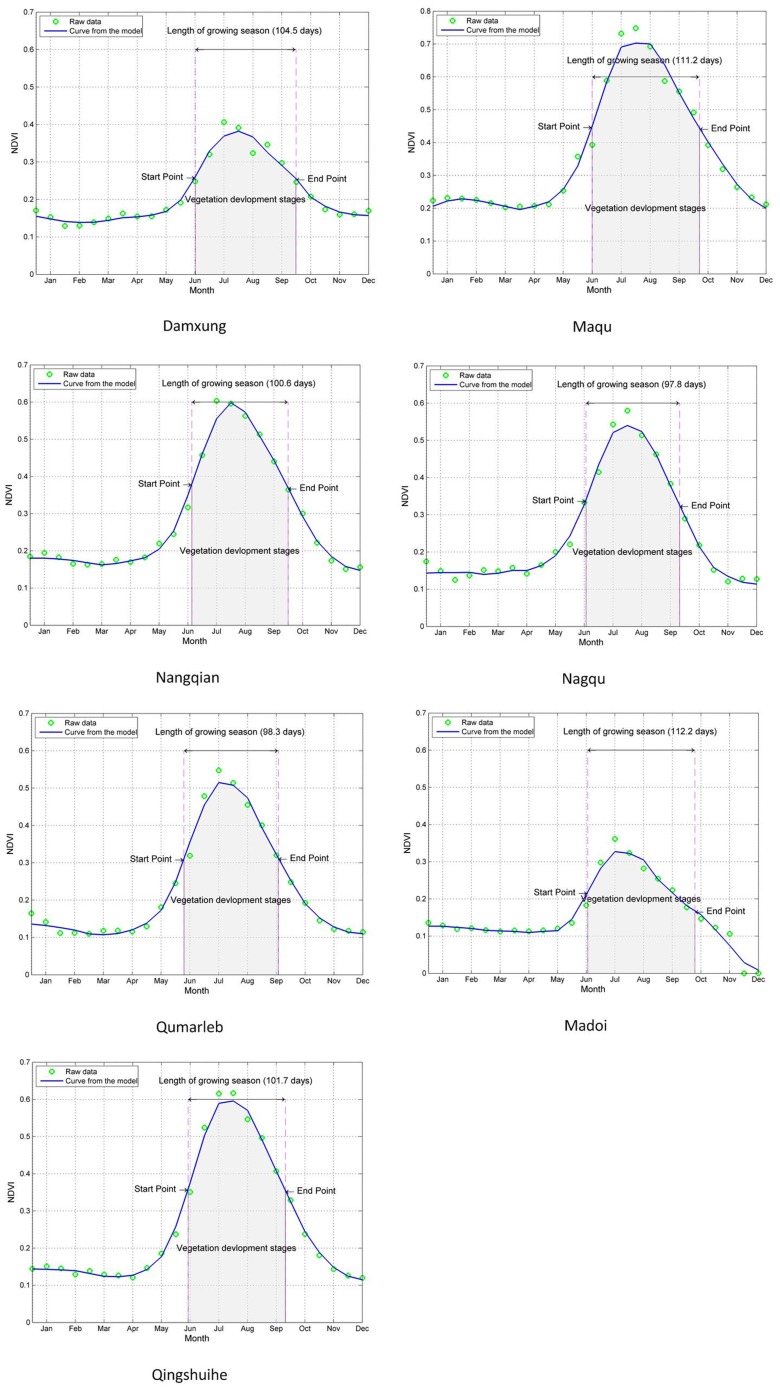
Growing season and related phenological measures for seven meteorological stations. The diagrams depict upper envelope Savitzky–Golay filtered data, with time in month steps. Dots represent original NDVI data. The thick solid lines show fitted functions of SG. The start point is defined as the date of the inflection point of the NDVI curve and end points as the date at which NDVI drops to the same level as the measured right minimum level. In between these dates, vegetation development stages are defined, covering the entire growing season (shaded area).

A set of analyses were initially performed to compare the effects on trends of maximum temperature and minimum temperature during growing seasons for the period 1982–2006 for the two groups (Fig. 5). It was found that although maximum temperature and minimum temperature both increased, the minimum temperature increased at a significantly faster pace in Group B than in Group A, resulting in a negative trend for the diurnal temperature range (DTR). For example, the normalized test statistic (Z) of DTR was −0.067, −0.018, −0.008 and −0.055 in the HLD stations of Nagqu, Maqu, Nangqian and Damxung, in contrast to 0.012, 0.027 and 0.045 in the stations with natural forces only, Qumarleb, Madoi and Qingshuihe, respectively (Table 5). The confidence in the trend for the Mann-Kendall statistic is calculated using a Kendall probability table [53]. By assessing the S result along with the number of samples, n, the Kendall table provides the probability of rejecting the null hypothesis (H0 = no trend) for a given level of significance. We calculate a ‘confidence level’ percentage by subtracting the probability (p) from 1 (Confidence = 1-p%). Confidence of 90% represents a significance level of α = 0.1, and 95% confidence corresponds to α = 0.05. The resulting confidence in the trend is applied in the Mann Kendall trend analysis as outlined in Table 3. The statistical probabilities of P-values were all above 95% except maximum temperature in Nangqian, and hence the null hypothesis (no trend) was rejected.

**Table 5:** Trend direction for the diurnal temperature range (DTR) using the Mann–Kendall (MK) test.

Group	Site Name	Maximum temperature					Minimum temperature					DTR	
		Mean Kendall Statistic (S)	Normalized Test Statistic (Z)	P-value	Result	Sen's slope	Mean Kendall Statistic (S)	Normalized Test Statistic (Z)	P-value	Result	Sen's slope	Slope	Trend Direction
***B***	***Nagqu***	***86***	***1.985***	***0.047***	***Trend exists***	***0.040***	***226***	***5.255***	***0.000***	***Trend exists***	***0.107***	***−0.067***	***Decreasing***
	***Maqu***	***162***	***3.760***	***0.000***	***Trend exists***	***0.066***	***170***	***3.947***	***0.000***	***Trend exists***	***0.084***	***−0.018***	***Decreasing***
	***Nangqian***	***84***	***1.939***	***0.053***	***Trend probably exists***	***0.057***	***144***	***3.340***	***0.001***	***Trend exists***	***0.065***	***−0.008***	***Decreasing***
	***Damxung***	***97***	***2.243***	***0.025***	***Trend exists***	***0.036***	***170***	***3.947***	***0.000***	***Trend exists***	***0.091***	***−0.055***	***Decreasing***
A	Qumarleb	140	3.246	0.001	Trend exists	0.084	126	2.919	0.004	Trend exists	0.072	0.012	Increasing
	Madoi	174	4.040	0.000	Trend exists	0.115	152	3.527	0.000	Trend exists	0.088	0.027	Increasing
	Qingshuihe	204	4.741	0.000	Trend exists	0.089	114	2.639	0.008	Trend exists	0.044	0.045	Increasing

DTR slopes are negative for all HLD stations (Group B), in contrast to the positive values for stations in areas of natural forces only (Group A). Note that the P-value is the statistical probability that the maximum or minimum temperature is increasing (Z>0) or decreasing (Z<0). The null hypothesis (no trend) is rejected for confidence above 90%. P-values above 95% mean the trend exists, P-values above 90% and below 95% mean the trend probably exists, and P-values below 90% mean there is no trend. It can be seen that the negative trend exists for all cases except the trend of maximum temperature in Nangqian.

**Figure 6:**
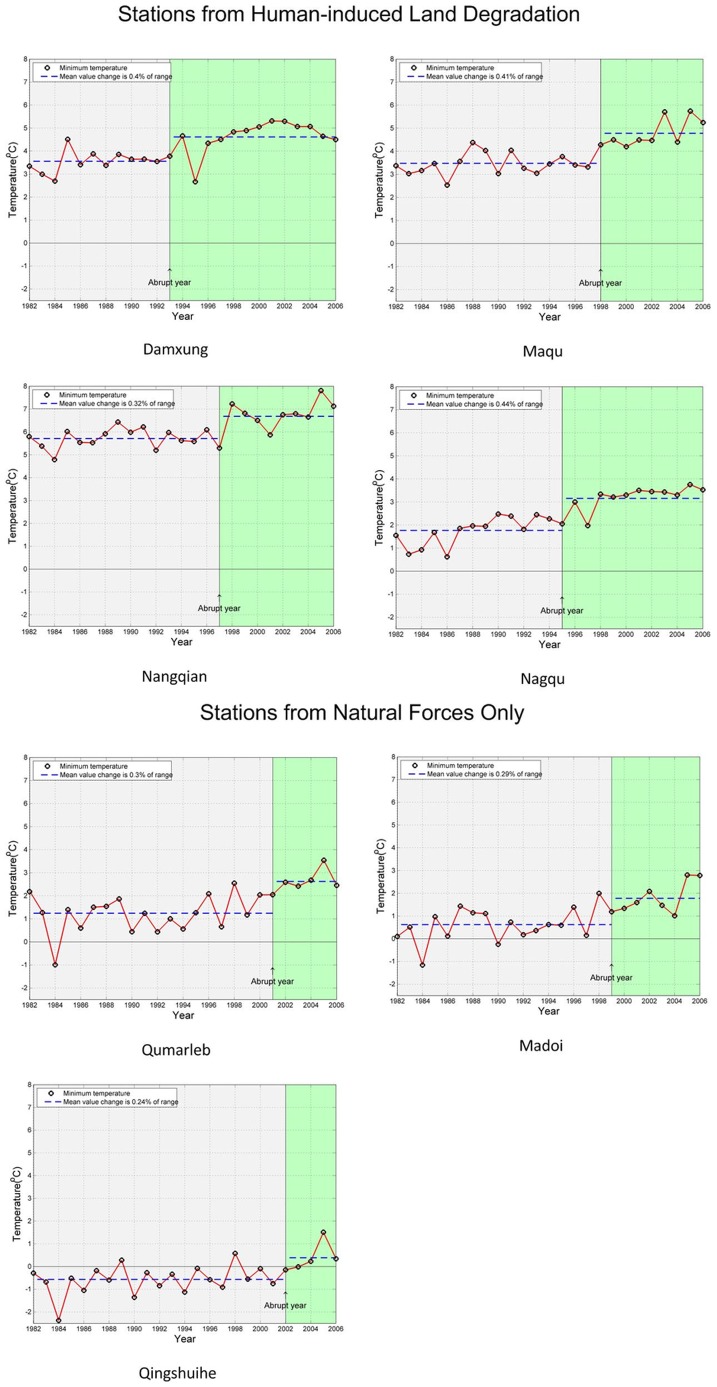
Comparison of abrupt year and amplitude of minimum temperature, tested by the M–K method. The abrupt year is the year determined to have an abrupt change within the timespan of 1982 to 2006. In HLD stations Nagqu, Maqu, Nangqian and Damxung the abrupt year was 1995, 1998, 1997 and 1993, respectively. It is clear that the abrupt year occurred earlier than at the stations in areas of natural forces only: 2001 for Qumarleb, 1999 for Madoi and 2002 for Qingshuihe. We also compared the amplitude change for each station. HLD stations exhibited a larger amplitude jump between the two periods divided by the abrupt year. For instance Nagqu, Maqu, Nangqian and Damxung changed by 0.44%, 0.41%, 0.32% and 0.4% respectively, compared to 0.3%, 0.29% and 0.24% in Qumarleb, Madoi and Qingshuihe.

Further analysis showed that there were abrupt changes for minimum temperature at all stations. However the year that abrupt changes happened was much earlier in Group B than in Group A, and the amplitude of this change was larger for Group B. The average abrupt year in Group B was about 5 years earlier: in 1996 vs. in 2001. The average amplitude change was 0.393% for Group B, about 1.4 times that (0.276%) for Group A (Fig. 6). These results reveal a significant and complex effect on the Tibet Plateau climate system from human activities, due to the multiple processes and feedbacks between degraded land surface from anthropogenic influences and the atmosphere. These results reveal differences between HLD and natural stations, which should be probed further in order to fully capture the role of human activities on the plateau climate system.

## Conclusions

There are some interesting implications of the differences between HLD stations and the stations in areas with natural forces only. Firstly, annual trends in minimum temperature in the HLD group increase at a much faster rate than in the group from areas with natural forces only, indicating that HLD has a strong impact on the observed temperature. This is consistent with the conclusion that persistent land degradation can notably increase temperatures [54], [55]. Secondly, the decreasing rate of annual DTR in the HLD group is particularly remarkable given that it is the reverse of the result from stations in areas with natural forces only. The result reinforces previous work suggesting that the reduction of vegetation cover and soil wetness may reduce the DTR by increasing nighttime surface air temperature [56]. This greater decrease may be partly attributed to (i) environmental conditions (e.g., vegetation) that reduce minimum temperature to a certain extent, thus minimizing the cooling mechanism in land degradation areas; (ii) weak evapotranspiration that prevents surface evaporative cooling effects in land degradation areas at very high-altitudes; and (iii) other factors that influence surface energy balance such as increased soil heating and long-wave surface forcing. These results demonstrate anthropogenic asymmetric changes to the climate, which reflect the complexity of the impact of human-caused land-cover change in the Earth's Third Pole. As such, more attention should be paid to issues such as HLD and more emphasis should be placed on characterizing and quantifying the effect of human-induced land-use change on asymmetric diurnal changes.

## References

[1] Marland G, Pielke RA Sr, Apps MJ, Avissar R, Betts RA, et al (2003) The climatic impacts of land surface change and carbon management, and the implications for climate-change mitigation policy. Clim Pol 3: 149–157.

[2] WMO (2005) Climate change and land degradation. WMO-No. 989, World Meteorological organization, Geneva, Switzerland. ISBN 92-63-10989-3. 6 p.

[3] Intergovernmental Panel on Climate Change (IPCC) (2007) The IPCC Fourth Assessment Report: Climate Change 2007: The Physical Science Basis, Cambridge Univ. Press, Cambridge, U. K. 2 p.

[4] Cornelis WM, Gabriels D (2009) Human-Induced Land Degradation. Land Use, Land Cover and Soil -Volume 3. ISBN: 978-1-84826-237-9.

[5] Davin EL, De Noblet-Ducoudré N (2010) Climatic impact of global-scale deforestation: radiative versus nonradiative processes. J Clim 23: 97–112.

[6] Rotenberg E, Yakir D (2010) Contribution of semi-arid forests to the climate system. Science 327: 451–454. 20093470 10.1126/science.1179998

[7] Lee X, Goulden ML, Hollinger DY, Barr A, Black TA, et al (2011) Observed increase in local cooling effect of deforestation at higher latitudes. Nature 479: 384–387. 22094699 10.1038/nature10588

[8] Karl TR, Jones PD, Knight RW, Kukla G, Plummer N, et al (1993) Asymmetric trends of daily maximum and minimum temperature. Bull Amer Met Soc 74: 1007–1023.

[9] Easterling DR, Horton B, Jones PD, Peterson TC, Karl TR, et al (1997) Maximum and minimum temperature trends for the globe. Science 277: 364–367.

[10] Folland CK, Karl TR (2001) Observed Climate Variability and Change. In: Houghton JT (Editor) Climate Change 2001: The Scientific Basis, Cambridge University Press, Cambridge. pp. 108–109.

[11] Jones PD, Groisman PYA, Coughlan M, Plummer N, Wang WC, et al (1990) Assessment of urbanization effects in time series of surface air temperature over land. Nature 347: 169–172.

[12] Gallo KP, Easterling DR, Peterson TC (1996) The Influence of Land Use/Land Cover on Climatological Values of the Diurnal Temperature Range. J Clim 9: 2941–2944.

[13] Gallo KP, Owen TW, Easterling DR, Jamason PF (1999) Temperature trends of the U.S. historical climatology network based on satellite-designated land use/land cover. J Clim 12: 1344–1348.

[14] Hale RC, Gallo KP, Loveland TR (2008) Influences of specific land use/land cover conversions on climatological normals of near surface temperature. J Geophys Res 113: D14113.

[15] Owen TW, Gallo KP, Elvidge CD, Baugh KE (1998) Using DMSP-OLS light frequency data to categorize urban environments associated with U.S. climate observing stations. Int J Remote Sens 19: 3451–3456.

[16] Spronken-Smith RA, Oke TR (1998) The thermal regime of urban parks in two cities with different summer climates. Int J Remote Sens 19: 2085–2104.

[17] Betts RA (2000) Offset of the potential carbon sink from boreal forestation by decreases in surface albedo. Nature 408: 187–190. 11089969 10.1038/35041545

[18] Owen TW, Gallo KP (2000) Updated population metadata for the United States Historical Climatology Network stations. J Clim 13: 4028–4033.

[19] Kalnay E, Cai M (2003) Impact of urbanization and land-use on climate change. Nature 423: 528–531. 12774119 10.1038/nature01675

[20] Pinker RT, Zhang B, Dutton EG (2005) Do Satellites Detect Trends in Surface Solar Radiation? Science 308: 850–854. 15879215 10.1126/science.1103159

[21] Barrow CJ (1991) Land Degradation: Development and Breakdown of Terrestrial Environments. Cambridge University Press, Cambridge.

[22] Hawkins TW, Brazel AJ, Stefanov WL, Bigler W, Saffell EM (2004) The role of rural variability in urban heat island determination for Phoenix, Arizona. J Appl Meteorol 43: 476–486.

[23] Fast JD, Torcolini JC, Redman R (2005) Pseudo-vertical temperature profiles and the urban heat island measured by a temperature data logger network in Phoenix. J Appl Meteorol 44: 3–13.

[24] White MA, Thornton PE, Running SW (1997) A continental phenology model for monitoring vegetation responses to interannual climatic variability. Glob Biogeochem Cyc 11: 217–234.

[25] Myneni RB, Tucker CJ, Asrar G, Keeling CD (1998) Interannual variations in satellite-sensed vegetation index data from 1981 to 1991. J Geophys Res 103 D6: 6145–6160.

[26] Zhou L, Tucker CJ, Kaufmann RK, Slayback D, Shabanov NV, et al (2001) Variations in northern vegetation activity inferred from satellite data of vegetation index during 1981 to 1999. J Geophys Res 106: 20069–20083.

[27] Pinzon J (2002) Using HHT to successfully uncouple seasonal and interannual components in remotely sensed data. SCI 2002, Conference Proceedings Jul 14–18, Orlando, Florida.

[28] Gallo KP (2005) Evaluation of temperature differences for paired stations of the U.S. Climate Reference Network. J Clim 18: 1631–1638.

[29] Aguilar E, Auer I, Brunet M, Peterson TC, Wieringa J (2003) Guidelines on Climate Metadata and Homogenization. WCDMP-No. 53, WMO-TD No. 1186, World Meteorological Organization, Geneva, Switzerland.

[30] Liu XY (2002) Past and Ongoing National, Provencial and Local Level Projects and Programs for Managing and Controlling Land Degradation by Using Participatory Approaches. Report for Asian Development Bank, People's Republic of China. 3544 p.

[31] IPCC, Watson RT, Zinyowera M, Moss RH (1996) Climate Change 1995, The IPCC Second Assessment Report. Cambridge University Press, Cambridge and New York. 862 p.

[32] Beniston M, Ohmura A, Wild M, Tschuck P, Rotach M (1996) Feedbacks between Mountains and Climate, Final Scientific Report Nr. 5001-035179 to the Swiss National Science Foundation, Bern, Switzerland.

[33] Ma Y, Wang Y, Wu R, Hu Z, Yang K, et al (2009) Recent advances on the study of atmosphere–land interaction observations on the Tibetan Plateau. Hydrol Earth Syst Sci 13: 1103–1111.

[34] Qiu J (2008) China: the third pole. Nature 454: 393–396. 18650887 10.1038/454393a

[35] Yao TD, Liu XD, Wang NL, Shi YF (2000) Amplitude of climatic changes in the Qinghai-Tibetan Plateau. Chin Sci Bull 45: 1236–1243.

[36] Dingguo Y (1992) Degradation and protection of grassland on the Qinghai-Tibet plateau. Erosion, Debris Flows and Environment in Mountain Regions. Proc Chengdu Symp. Jul 1992 IAHS Pub no. 209: 471–476.

[37] Jönsson P, Eklundh L (2004) TIMESAT- A Program for Analyzing Time-series of Satellite Sensor Data. Comp Geosci 30: 833–845.

[38] Brown ME, Pinzon JE, Tucker CJ (2005) Quantitative comparison of four AVHRR global data sets for land applications. NASA Global Mapping and Modell. Syst. (GIMMS), NASA Goddard Space Flight Cent., Greenbelt, Md. 35 p.

[39] Tucker CJ, Pinzon JE, Brown ME, Slayback D, Pak EW, et al (2005) An Extended AVHRR 8-km NDVI Data Set Compatible with MODIS and SPOT Vegetation NDVI Data. INT J REMOTE SENS 26: 4485–4498.

[40] Lin ZY, Wu XD (1981) Climatic regionalization of the Qing-hai-Xizang Plateau. Acta Geogr Sin 36: 22–32.

[41] Gilbert RO (1987) Statistical Methods for Environmental Pollution Monitoring. Van Nostrand Reinhold, New York, NY, ISBN 0-442-23050-8.

[42] Tabari H, Marofi S, Ahmadi M (2011) Long-term variations of water quality parameters in the Maroon River. Iran. Env Mon Assess 177: 273–287. 10.1007/s10661-010-1633-y 20700652

[43] Chattopadhyay S, Jhajharia D, Chattopadhyay G (2011) Univariate modelling of monthly maximum temperature time series over northeast India: neural network versus Yule–Walker equation based approach. Meteorol Appl 18: 70–82.

[44] Modarres R, Silva VPR (2007) Rainfall trends in arid and semi-arid regions of Iran. J Arid Environ 70: 344–355.

[45] Theil H (1950) A rank-invariant method of Linear and polynomial regression analysis. I, II, III, Nederl Akad Wetensch Proc 53: 386–392, 512–525, 1397–1412.

[46] Sen PK (1968) Estimates of the regression coefficient based on Kendall's tau. J Am Stat Assoc 39: 1379–1389.

[47] Goossens C, Berger A (1987) How to recognize an abrupt climate change? In: Berger WH, Labeyrie LD (eds) Abrupt climate change, evidence and implications. NATO ASI series C: Mathematical and Physical Sciences, Vol. 216: . pp. 31–46.

[48] Gerstengarbe FW, Werner PC (1999) Estimation of the beginning and end of recurrent events within a climate regime. Clim Res 11: 97–107.

[49] Lorenc AC, Hammon O (1988) Objective quality control of observations using Bayesian methods. Theory, and a practical implementation. Q J R Meteorol Soc 114: 515–543.

[50] Andersson E, Järvinen H (1999) Variational quality control. Q J R Meteorol Soc 125: 697–722.

[51] Feng S, Hu Q, Qian WH (2004) Quality control of daily meteorological data in China, 1951–2000: A new dataset. INT J CLIMATOL 24: 853–870.

[52] Wessels KJ, Prince SD, Malherbe J, Small J, Frost PE, et al (2007) Can human-induced land degradation be distinguished from the effects of rainfall variability? A case study in South Africa. J ARID ENVIRON 68: 271–297.

[53] Hollander M, Wolfe DA (1973) Nonparametric Statistical Methods. Wiley, New York, USA.

[54] Balling RC (1991) Impact of Desertification on Regional and Global Warming. Bull Amer Meteorol Soc 72: 232–23.

[55] Balling RC Jr, Klopatek JM, Hildebrandt ML, Moritz CK, Watts CJ (1998) Impacts of land degradation on historical temperature records from the Sonoran desert. Clim Ch 40: 669–681.

[56] Zhou L, Dickinson RE, Tian Y, Vose RS, Dai Y (2007) Impact of vegetation removal and soil aridation on diurnal temperature range in a semiarid region: Application to the Sahel. Proc Natl Acad Sci U.S.A 104 46: 17937–17942. 17986620 10.1073/pnas.0700290104 PMC2084275

[57] Andersson E, Brogaard S, Olsson L (2011) The Political Ecology of Land Degradation. Ann Rev Env Res 36: 295–319.

